# Growth differentiation factor 15 ameliorates nonalcoholic steatohepatitis and related metabolic disorders in mice

**DOI:** 10.1038/s41598-018-25098-0

**Published:** 2018-05-01

**Authors:** Kook Hwan Kim, Seong Hun Kim, Dai Hoon Han, Young Suk Jo, Yong-ho Lee, Myung-Shik Lee

**Affiliations:** 1 Severance Biomedical Research Institute, Yonsei University College of Medicine, 50-1 Yonsei-ro, Seodaemun-gu, Seoul, 03722 South Korea; 2 Department of Health Sciences and Technology, SAIHST, Sungkyunkwan University, 81 Irwon-ro, Gangnam-gu, Seoul, 06351 South Korea; 3 Department of Surgery, Yonsei University College of Medicine, 50-1 Yonsei-ro, Seodaemun-gu, Seoul, 03722 South Korea; 4 Department of Internal Medicine, Yonsei University College of Medicine, 50-1 Yonsei-ro, Seodaemun-gu, Seoul, 03722 South Korea

## Abstract

Growth differentiation factor 15 (GDF15) is an endocrine hormone belonging to the TGFβ superfamily member. GDF15 administration or GDF15 overexpression has been reported to have anti-obesity and anti-diabetic effects. Although non-alcoholic fatty liver disease (NAFLD)/non-alcoholic steatohepatitis (NASH) is frequently associated with obesity and insulin resistance, the functional role of endogenous GDF15 and therapeutic effect of GDF15 overexpression in NASH and related metabolic deterioration have not been evaluated. Here, we found that GDF15 expression was increased in the livers of NASH animal models and human subjects with NASH. Elevated expression of GDF15 was due to diet-induced hepatic endoplasmic reticulum (ER) stress. *Gdf15*-knockout mice exhibited aggravated NASH phenotypes such as increased steatosis, hepatic inflammation, fibrosis, liver injury, and metabolic deterioration. Furthermore, GDF15 directly suppressed expression of fibrosis-related genes and osteopontin (OPN), contributing factors for NASH-related fibrosis, in hepatic stellate cells *in vitro* and in the liver of mice *in vivo*. Finally, we found that *GDF15*-transgenic mice showed attenuation of NASH phenotypes and metabolic deterioration. Therefore, our results suggest that induction of endogenous GDF15 is a compensatory mechanism to protect against the progression of NASH and that GDF15 could be an attractive therapeutic candidate for treatment of NASH and NASH-related metabolic deterioration.

## Introduction

Non-alcoholic fatty liver disease (NAFLD) is one of the most common chronic liver diseases worldwide^1^. NAFLD encompasses a pathological spectrum of liver diseases ranging from simple steatosis to non-alcoholic steatohepatitis (NASH). NASH is defined as hepatic steatosis with inflammation and fibrosis in the liver, and can progress to more severe stages such as cirrhosis and hepatocellular carcinoma (HCC)^2^. NAFLD/NASH is frequently accompanied by obesity, dyslipidemia or type 2 diabetes, and thereby NAFLD/NASH patients typically have obesity, insulin resistance or diabetes^3^. Simple steatosis can lead to the development of NASH accompanied by inflammation or fibrosis through multiple events including insulin resistance, oxidative stress, endoplasmic reticulum (ER) stress, mitochondrial dysfunction, adipose tissue dysfunction, altered regulation of innate immunity or compositional changes of microbiota^4^. Based on these findings, the “multi-parallel hit” hypothesis has been recently proposed to explain the pathogenesis of NASH. Despite growing number of multiple therapeutic targets, there are currently no approved pharmacological drugs for treatment of NASH.

Growth differentiation factor 15 (GDF15) belongs to the transforming growth factor beta (TGFβ) superfamily member and is also known as NAG-1 (nonsteroidal anti-inflammatory drug-activated gene-1), MIC-1 (macrophage inhibitory cytokine-1), PLAB (placental bone morphogenetic protein) or PTGFB (placental transforming growth factor beta)^5–9^. GDF15 is widely expressed in various tissues with the highest levels in the liver and placenta^5,8,9^. Numerous studies suggest that GDF15 is induced by various stimuli such as fatty acids or mitochondrial stress^10,11^. Moreover, GDF15 is implicated in aging and in the development of aging-related pathological diseases such as obesity, cardiovascular diseases, diabetes or cancer^12^. It has been reported that serum/plasma GDF15 levels are increased in human subjects with diverse diseases such as cancer^13^, diabetes^14^, mitochondrial disorders^15^, cardiac failures^16^, renal failures^17^ and chronic liver diseases (chronic hepatitis, cirrhosis and HCC)^18,19^. In addition, recent paper suggest that serum GDF15 level is also increased in human NASH subjects with advanced fibrosis^20^. These results suggest that GDF15 could be used as a potential diagnostic biomarker for diverse diseases.

Several studies suggest that exogenous GDF15 exerts protective effects against obesity and insulin resistance^11,21,22^, although the role of endogenous GDF15 in these diseases has not been clearly shown. However, little is known regarding to the functional role of endogenous GDF15 and therapeutic effect of GDF15 overexpression in NASH. Here, we observed elevated GDF15 expression in the liver of two different dietary mouse models of NASH and human subjects with NASH. We also showed aggravated hepatic steatosis, inflammation, fibrosis and metabolic deterioration in *Gdf15*-knockout (*Gdf15*^−/−^) mice fed two different NASH diets, while these pathologic and metabolic features were attenuated in *GDF15*-transgenic (*GDF15*-Tg) mice. These results suggest that GDF15 is induced in the liver as a compensatory mechanism to protect against NASH and related metabolic disorders.

## Results

### GDF15 is upregulated in the livers of mice and human subjects with NASH

**Figure 1:**
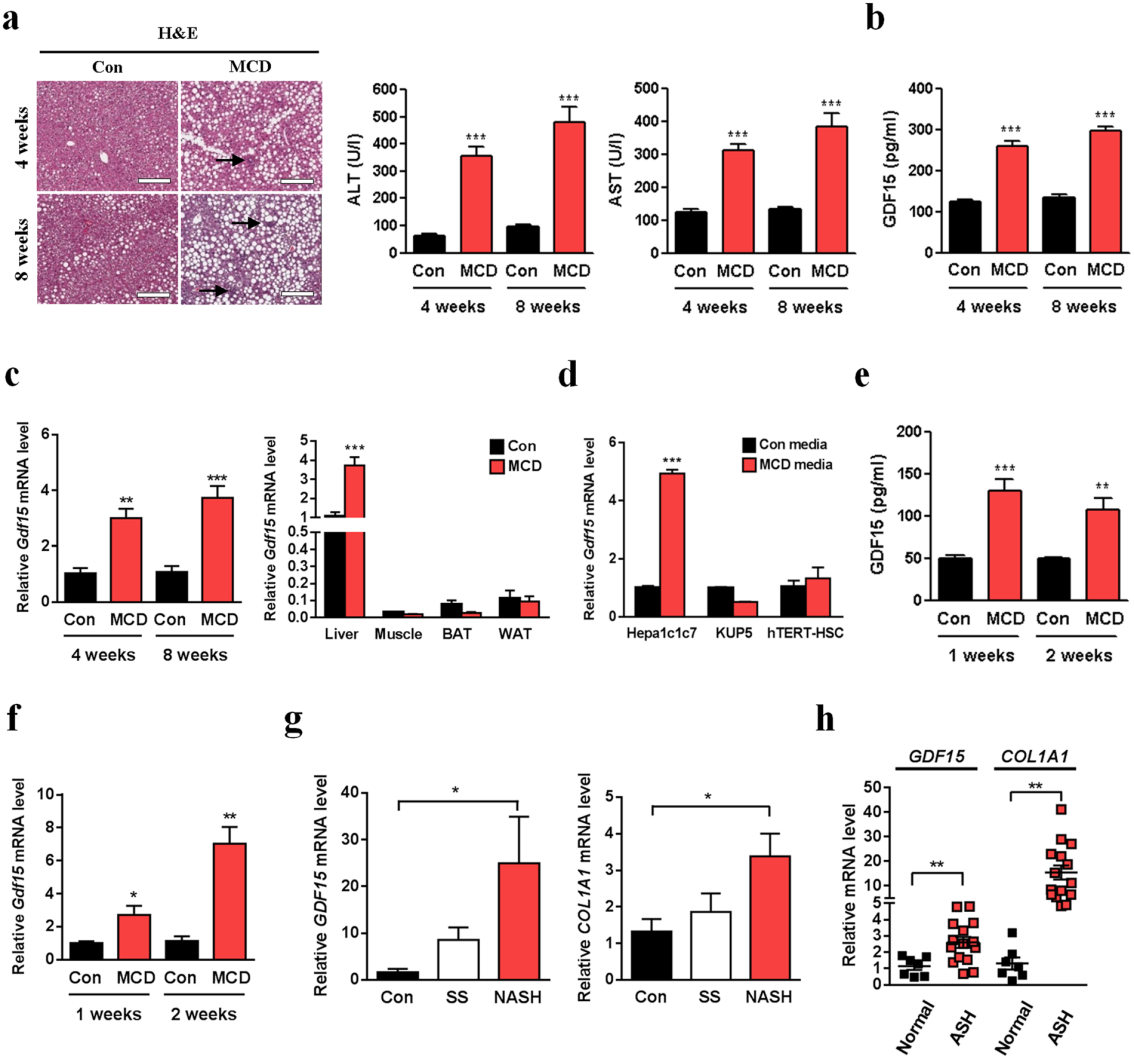
GDF15 is induced in the livers of MCD diet-fed mice and human NASH subjects. (**a**) Liver H&E staining and serum ALT/AST levels (n = 8) in C57BL/6 mice fed MCD diet for 4 or 8 weeks. Scale bar, 200 μm. Arrows indicate inflammatory loci. (**b**) Serum GDF15 level in C57BL/6 mice fed MCD diet for 4 or 8 weeks (n = 6–8). (**c**) Relative hepatic *Gdf15* mRNA level in C57BL/6 mice fed MCD diet for 4 or 8 weeks (left panel, n = 3–5), and relative *Gdf15* mRNA level in various metabolic organs in mice fed MCD diet for 8 weeks (right panel, n = 5). (**d**) Relative *Gdf15* mRNA levels in Hepa1c1c7, KUP5 or hTERT-HSC cell lines incubated in MCD media for 18 h (n = 5). (**e,f**) Serum GDF15 (**e**, n = 5) and relative hepatic *Gdf15* mRNA levels (**f**, n = 4–5) in mice fed MCD diet for 1 or 2 weeks. (**g**) Relative *GDF15* and *COL1A1* mRNA levels in the livers from control human subjects and subjects with simple steatosis (SS) or NASH (n = 6). (**h**) Relative *GDF15* and *COL1A1* mRNA levels in the liver tissue from subjects with alcoholic steatohepatitis (ASH, GSE28619) (control, n = 7; ASH, n = 15). Data are means ± SEM. *p < 0.05, **p < 0.01, ***p < 0.001.

To analyze the change of hepatic or serum GDF15 expression in NASH animal models, we employed a methionine-choline-deficient (MCD) diet model, a widely-used dietary NASH model. When fed MCD diet for 4 or 8 weeks, mice showed increased hepatic lipid accumulation and inflammation (Fig. 1a), while whole body and liver weights were reduced (data not shown). Serum alanine aminotransferase (ALT) or aspartate aminotransferase (AST) concentrations were also elevated in MCD diet-fed mice (Fig. 1a). Interestingly, we observed significantly increased serum GDF15 level in mice fed MCD diet for 4 or 8 weeks compared to control diet-fed mice (Fig. 1b). Moreover, *Gdf15* gene expression was increased in the liver but not in skeletal muscle, brown adipose tissue or white adipose tissue (Fig. 1c), suggesting that increased liver GDF15 expression might contribute to the increase of systemic GDF15 level in MCD diet-fed mice. Consistent with a previous report that GDF15 expression was detected only in parenchymal cells but not in non-parenchymal cells in the liver^5^, *Gdf15* expression was increased only in a hepatocyte cell line but not in a hepatic stellate cell (HSC) line or a Kupffer cell line after exposure to MCD media in an *in vitro* model of NASH (Fig. 1d), suggesting that increased GDF15 expression in hepatocytes might contribute to the increases of hepatic *Gdf15* expression and serum GDF15 level in MCD diet-induced NASH. Serum level and hepatic expression of GDF15 were also elevated in mice fed MCD diet for only 1 week or 2 weeks (Fig. 1e,f), suggesting that short-term feeding of MCD diet is sufficient to induce GDF15 expression.

In addition to the results of mouse liver tissues, *GDF15* expression was significantly higher in the liver of patients with NASH compared to that of control subjects (Fig. 1g). Moreover, microarray transcriptome data sets of alcoholic steatohepatitis (ASH)^23^ revealed that *GDF15* expression was elevated in the liver of human ASH patients compared to control subjects (Fig. 1h), suggesting that GDF15 is a potential biomarker for a variety of steatohepatitis. Taken together, these results suggest that GDF15 expression is induced in the livers of both mice and humans with NASH.

### MCD diet-induced GDF15 expression is due to ER stress

**Figure 2:**
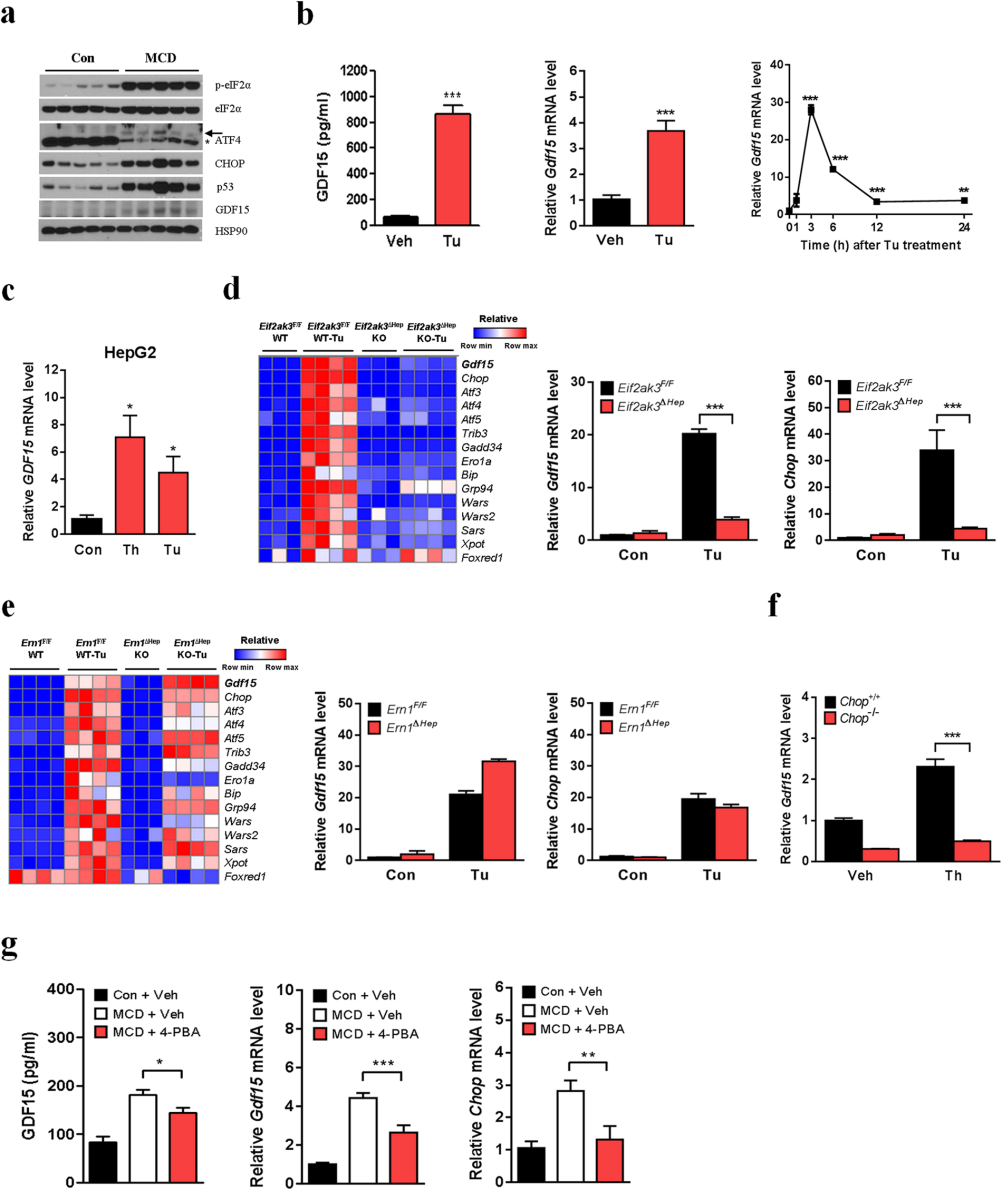
ER stress mediates MCD diet-induced GDF15 expression. (**a**) Immunoblot analysis of liver lysates from C57BL/6 mice fed MCD diet for 8 weeks. Asterisk indicates a non-specific band. (**b**) Serum GDF15 (left panel, n = 6) and relative hepatic *Gdf15* mRNA levels (middle panel, n = 4) in C57BL/6 mice 24 h after tunicamycin (Tu, 1 mg/kg) or vehicle (Veh) injection. Time-course of the changes in mouse liver *Gdf15* mRNA level after Tu injection (right panel, n = 3). (**c**) Relative *GDF15* mRNA level in HepG2 cells after incubation with Tu (5 μg/ml) or thapsigargin (Th, 1 μM) for 24 h (n = 3). (**d**,**e**) A heatmap showing relative expression of *Gdf15* and upstream or downstream genes of CHOP, and *Gdf15/Chop* mRNA levels in the livers of *Eif2ak3*^Δhep^ mice (**d**, GSE29929) and *Ern1*^Δhep^ mice (**e**, GSE40515). (**f**) Relative *Gdf15* mRNA level in *Chop*^–/–^ MEFs treated with Th (1 μM) (n = 3). (**g**) Serum GDF15 (n = 8–9) and relative hepatic *Gdf15* or *Chop* mRNA levels (n = 5) in MCD diet-fed C57BL/6 mice treated with 4-PBA (1 g/kg/day) or vehicle (Veh) for 9 days. Data are means ± SEM. *p < 0.05, **p < 0.01, ***p < 0.001.

We next investigated the molecular mechanisms underlying GDF15 induction in MCD diet-induced NASH. Since it has been reported that p53 plays an important role in pathogenesis of MCD diet-induced NASH^24^, and that p53 regulates GDF15 expression^25^, we studied the involvement of p53 in GDF15 induction by MCD diet. Consistent with the increased *Gdf15* mRNA level, GDF15 protein level was also elevated in the livers after MCD diet feeding (Fig. 2a). p53 was also remarkably induced in the livers of MCD diet-fed mice (Fig. 2a), in line with a previous report^24^. However, the p53 inhibitor, pifithrin-α did not inhibit MCD diet-induced *Gdf15* expression (Supplementary Fig. 1a,b), suggesting that p53 is not important in hepatic GDF15 induction by MCD diet. Since ER stress has been implicated in the pathogenesis of NASH^26^, we studied the contribution of ER stress to MCD diet-induced GDF15 expression. MCD diet caused increased expression of ER stress marker proteins, phosphorylated eIF2α, ATF4 and CHOP (also known as DDIT3) in the livers (Fig. 2a). When we administered a well-known ER stressor, tunicamycin to mice, serum GDF15 level and hepatic *Gdf15* expression were higher than those of vehicle-treated mice (Fig. 2b). Treatment of HepG2 cells with ER stressors such as tunicamycin or thapsigargin also induced *GDF15* expression (Fig. 2c), suggesting that ER stress induces hepatic GDF15 expression *in vitro* as well as *in vivo*.

To evaluate the molecular mechanism underlying *in vivo* GDF15 induction by ER stress, we analyzed transcriptome data sets of livers from mice with hepatic deletion of *eukaryotic translation initiation factor 2 alpha kinase 3* (*Eif2ak3*, also known as *Perk*)^27^ or *ER to nucleus signalling 1* (*Ern1*, also known as *Ire1a*) gene^28^. Tunicamycin-induced *Gdf15* expression was markedly lower in the liver of hepatocyte-specific *Eif2ak3*-knockout (*Eif2ak3*^Δhep^) mice compared to wild-type mice (Fig. 2d), while there was no difference in GDF15 expression between hepatocyte-specific *Ern1*-knockout (*Ern1*^Δhep^) and wild-type mice (Fig. 2e). Furthermore, heatmap analysis showed that the pattern of *Gdf15* expression in *Eif2ak3*^Δhep^ mice and wild-type mice mirrored that of CHOP target genes such as *Trib3*, *Gadd34* (also known as *Ppp1r15a*), *Sars*, *Wars* or *Xpot* (Fig. 2d). *Chop* induction after tunicamycin treatment was also significantly lower in the livers of *Eif2ak3*^Δhep^ mice but not in the livers of *Ern1*^Δhep^ mice compared to their respective control mice (Fig. 2d,e). *Gdf15* induction by ER stressor was also much lower in *Chop*^−/−^ mouse embryonic fibroblasts (MEFs) compared to *Chop*^+/+^ MEFs (Fig. 2f). These results suggest that PERK-CHOP mediates ER stress-induced GDF15 expression. To study whether ER stress directly mediates MCD diet-induced GDF15 expression, we administrated a chemical ER chaperone, 4-phenylbutyric acid (4-PBA) to mice. As hypothesized, MCD diet-fed mice treated with 4-PBA showed reduced serum GDF15 level and hepatic *Gdf15* expression compared to vehicle-treated MCD diet-fed mice, which was accompanied by reduced hepatic *Chop* expression (Fig. 2g). Taken together, our results suggest that ER stress contributes to hepatic GDF15 induction in MCD-induced NASH.

### Gdf15 deletion aggravates MCD diet-induced NASH in mice

**Figure 3:**
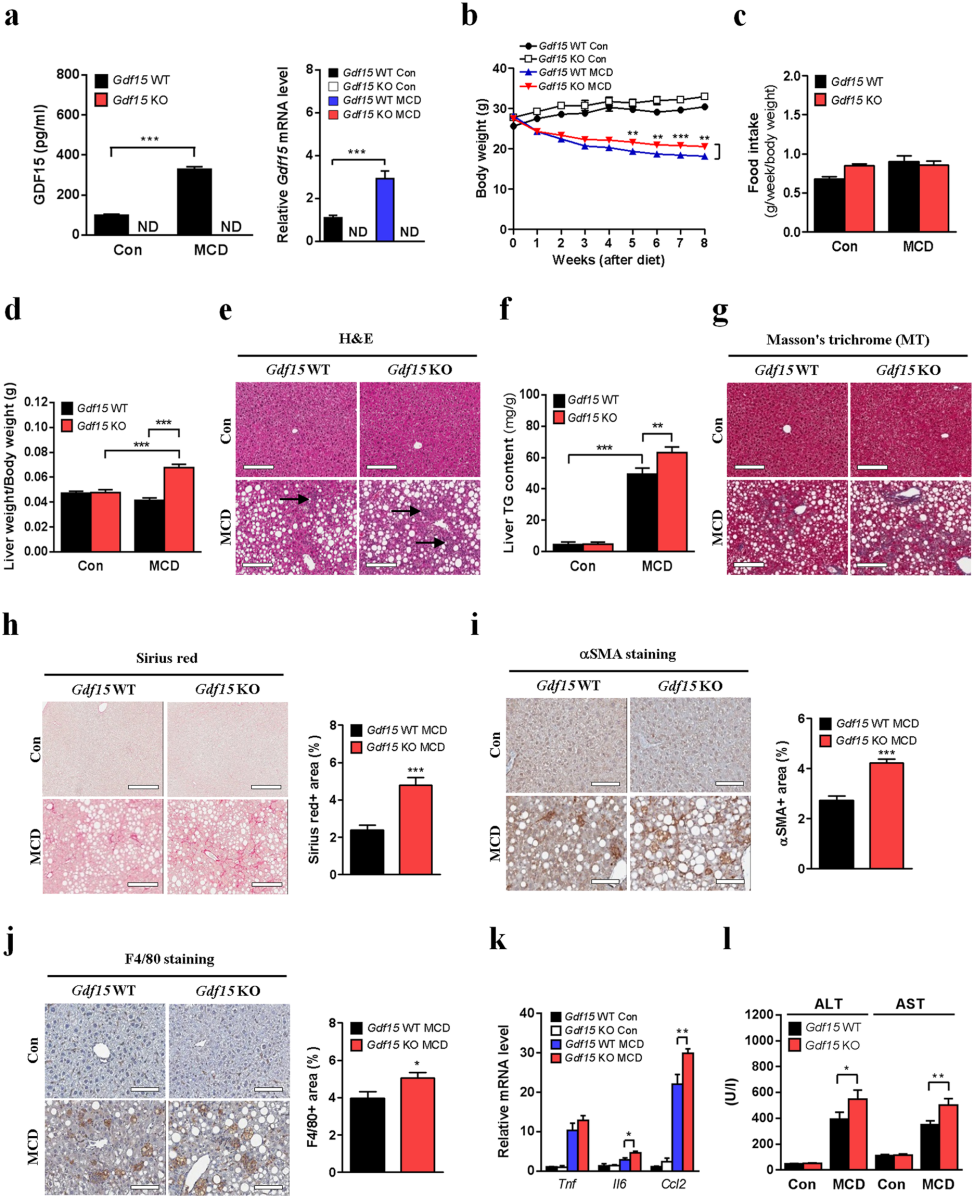
*Gdf15* deletion accelerates NASH in mice fed MCD diet for 8 weeks. (**a**) Serum GDF15 level (left panel, n = 5–7) and relative hepatic *Gdf15* mRNA level (right panel, n = 3–4). ND indicates not detected. (**b**) Body weight (n = 5–7). (**c**) Food intake adjusted for body weight (n = 5–7). (**d)** Liver weight adjusted for body weight (n = 5–7). (**e**) Liver H&E staining. Arrows indicate inflammatory loci. (**f**) Liver TG content (n = 5–7). (**g)** Masson’s trichrome (MT) staining. (**h)** Sirius red staining and quantification. (**i**) αSMA immunostaining and quantification. (**j)** F4/80 immunostaining and quantification. (**k**) Relative inflammatory gene expression (n = 3–4). (**l**) Serum ALT/AST levels (n = 5–7). Scale bars for H&E, MT and Sirius red staining, 200 μm. Scale bars for αSMA and F4/80 immunostaining, 100 μm. Data are means ± SEM. *p < 0.05, **p < 0.01, ***p < 0.001.

To study the role of GDF15 induction in MCD diet-induced NASH, we challenged *Gdf15*^−/−^ mice with MCD diet. As expected, neither serum GDF15 level nor hepatic *Gdf15* expression was increased in *Gdf15*^−/−^ mice after MCD diet feeding (Fig. 3a). The decrease of body weight caused by MCD diet was significantly less in *Gdf15*^−/−^ mice compared to wild-type mice (Fig. 3b), while food intake was not different between the two groups (Fig. 3c). Intriguingly, loss of liver weight after MCD diet was also significantly less in *Gdf15*^−/−^ mice compared to control mice (Supplementary Fig. 2a). Liver weight adjusted for body mass was not decreased but increased in *Gdf15*^−/−^ mice after MCD diet feeding (Fig. 3d), implying that GDF15 modulates liver size in NASH.

When we conducted histologic analysis to compare the progression of NASH between *Gdf15*^−/−^ and wild-type mice, the number of inflammatory loci and the size of lipid droplets were apparently increased in the liver of *Gdf15*^−/−^ mice compared to that of control mice after MCD diet feeding (Fig. 3e). As expected, hepatic triglyceride (TG) content was also increased in MCD diet-fed *Gdf15*^−/−^ mice (Fig. 3f). This change was probably due to the reduced expression of β-oxidation-related genes (*Acadl* and *Acadm*) (Supplementary Fig. 2b), while the expression of fatty acid synthesis-related genes was not altered (Supplementary Fig. 2c). Furthermore, exogenous GDF15 treatment slightly but significantly increased the expression of several β-oxidation-related genes in primary hepatocytes but not that of fatty acid synthesis-related genes, fatty acid uptake-related genes or lipolytic genes (Supplementary Fig. 2d), suggesting that increased hepatic lipid accumulation by GDF15 deletion is probably due to impaired β-oxidation. Additionally, Masson’s trichrome and Sirius red staining showed aggravated fibrosis in the liver of MCD diet-fed *Gdf15*^−/−^ mice (Fig. 3g,h). In parallel, *Gdf15*^−/−^ mice had a significantly increased number of alpha-smooth muscle actin (αSMA, also known as ACTA2)-positive cells in the liver (Fig. 3i). Furthermore, the number of F4/80 (also known as ADGRE1)-positive macrophages was increased in the liver of MCD diet-fed *Gdf15*^−/−^ mice compared to MCD diet-fed control mice (Fig. 3j), which was accompanied by elevated expression of inflammatory genes (*Il6*, *Ccl2*) in the liver (Fig. 3k). In contrast, the number of Kupffer cells positive for C-type lectin domain family 4, member f (CLEC4F), a specific-Kupffer cell marker^29^ and *Clec4f* mRNA expression were not different between *Gdf15*^−/−^ and control mice fed MCD diet (Supplementary Fig. 2e,f), suggesting that infiltration of monocyte-derived macrophages is increased in the liver of *Gdf15*^−/−^ mice probably due to increased expression of chemoattractant cytokine such as CCL2. Additionally, serum ALT/AST levels were increased in MCD diet-fed *Gdf15*^−/−^ mice compared to MCD diet-fed controls (Fig. 3l). Taken together, these results indicate that endogenous GDF15 induction is a compensatory mechanism to protect against NASH and liver damage caused by MCD diet feeding.

### Gdf15 deletion accelerates AMLN diet-induced NASH and metabolic deterioration in mice

Although MCD diet is widely used to induce NASH in mice, this model has limited value as a model for human NASH. Of note, it has been reported that MCD diet-fed mice exhibit remarkable weight loss, low fasting glucose and insulin levels, reduced cholesterol and TG levels or increased insulin sensitivity^30^, which are in contrast to those in human subjects with NASH. Thus, we employed Amylin liver NASH (AMLN) diet model as a physiologically relevant dietary model for human NASH. AMLN diet-fed mice have been recently reported to show metabolic characteristics similar to those of human NASH subjects^31^. To confirm the effect of AMLN diet on NASH progression in mice, we fed C57BL/6 mice with standard chow diet (Lab Diet 5053), AMLN diet or matched control diet for 30 weeks. AMLN diet-fed mice exhibited aggravation of NASH phenotypes and related metabolic deterioration compared to mice fed matched control diet or standard chow diet (Supplementary Fig. 3a–l). Similar results were also observed in C57BL/6 mice fed AMLN diet for 22 weeks (data not shown). Since small but significant differences in almost all metabolic parameters were observed between mice fed standard chow diet and those fed matched control diet (Supplementary Fig. 3a–l), we employed mice fed AMLN diet and those fed matched control diet for all further studies.

**Figure 4:**
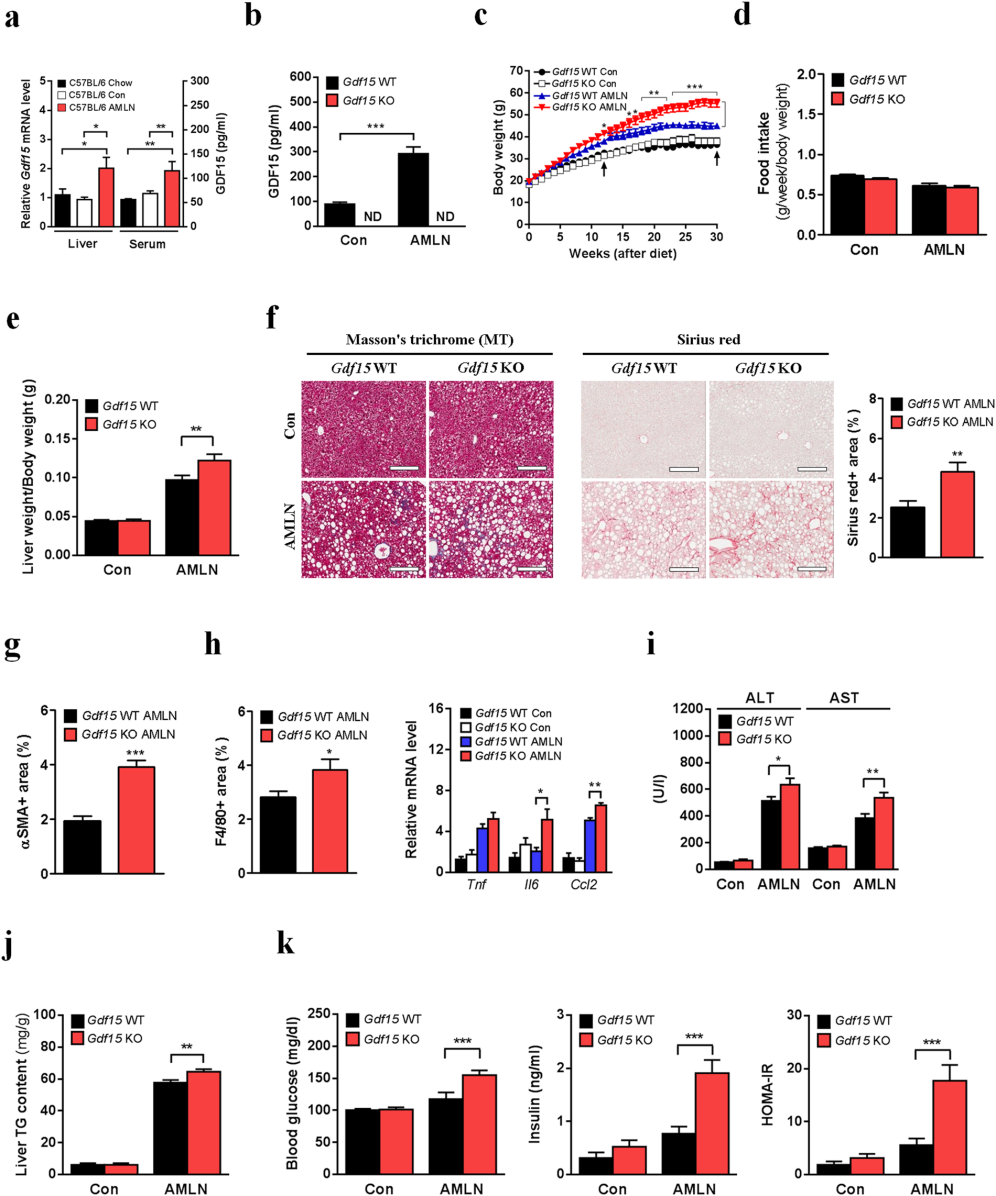
*Gdf15* deletion aggravates NASH and related metabolic parameters in AMLN diet-fed mice. (**a**) Relative hepatic *Gdf15* mRNA (n = 5–7) and serum GDF15 levels (n = 7) in C57BL/6 mice fed standard chow (Chow), matched control (Con) or AMLN diet (AMLN). (**b**–**k**) Serum GDF15 level (**b**, n = 6–10), body weight (**c**, n = 7–16), food intake adjusted for body weight (**d**, n = 7–12), liver weight adjusted for body weight (**e**, n = 7–12), liver MT staining (left panel of **f**), Sirius red staining and quantification (right panel of **f**), quantification of αSMA immunochemistry (**g**), quantification of F4/80 immunochemistry (left panel of **h**), relative inflammatory gene expression (right panel of **h**, n = 5–7), serum ALT/AST levels (**i**, n = 6–9), liver TG content (**j**, n = 6–9), fasting glucose/insulin levels (left and middle panels of **k**, n = 4–6) and HOMA-IR index (right panel of **k**, n = 4–6) in *Gdf15*^–/–^ and control mice fed AMLN or control diet for 30 weeks. ND indicates not detected. Arrows indicate the time of body weight measurement at 12 weeks and 30 weeks of AMLN diet feeding. Scale bars for MT and Sirius red staining, 200 μm. Data are means ± SEM. *p < 0.05, **p < 0.01, ***p < 0.001.

In line with the results from MCD diet model, hepatic *Gdf15* expression and serum GDF15 level were increased in AMLN diet-fed C57BL/6 or *Gdf15*^+/+^ mice compared to control diet-fed mice (Fig. 4a,b and Supplementary Fig. 4a). Intriguingly, *Gdf15*^–/–^ mice fed AMLN diet for 30 weeks gained more body and liver weights compared to control mice (Fig. 4c and Supplementary Fig. 4b,c) without difference in food intake (Fig. 4d), while there was no difference in body weight between *Gdf15*^−/−^ and control mice fed matched control diet (Fig. 4c). Liver weight adjusted for body mass was also increased in AMLN diet-fed *Gdf15*^−/−^ mice (Fig. 4e), supporting that GDF15 functions as an inhibitory factor against liver hypertrophy, similar to the results of MCD diet model.

When we conducted histologic analysis to compare the progression of NASH between *Gdf15*^−/−^ and wild-type mice fed AMLN diet, we observed aggravated fibrosis (Fig. 4f,g and Supplementary Fig. 4d), increased infiltration of F4/80-positive cells (Fig. 4h and Supplementary Fig. 4e,f), increased expression of inflammatory cytokines (Fig. 4h) and increased serum ALT/AST levels (Fig. 4i) in *Gdf15*^−/−^ mice compared to control mice fed AMLN diet for 30 weeks. However, the numbers of CLEC4F-positive Kupffer cells were not different between the two groups fed AMLN diet (Supplementary Fig. 4g), supporting that infiltration of monocyte-derived macrophages into the liver of AMLN diet-fed mice is modulated by GDF15. Liver TG content of *Gdf15*^−/−^ mice was also slightly but significantly higher than that of control mice after AMLN diet feeding for 30 weeks (Fig. 4j). The difference in TG content between *Gdf15*^−/−^ and control mice was more pronounced after AMLN diet feeding for 12 weeks compared to feeding for 30 weeks (Supplementary Fig. 4h), suggesting that ongoing lipid accumulation might mask the difference in the lipid accumulation during a long-term feeding of AMLN diet. Although the difference in body weight between *Gdf15*^−/−^ and control mice was less pronounced after AMLN diet feeding for 12 weeks compared to feeding for 30 weeks (Fig. 4c), *Gdf15*^−/−^ mice had significantly higher liver weight adjusted for body mass and serum ALT/AST levels after 12 weeks of AMLN diet feeding (Supplementary Fig. 4i,j), suggesting that GDF15 acts as an inhibitory factor against liver hypertrophy and could play a protective role against NASH regardless of body weight changes. Intriguingly, *Gdf15*^−/−^ mice fed AMLN diet for 30 weeks also had increased fasting glucose/insulin levels and homeostatic model assessment of insulin resistance (HOMA-IR) index compared to control mice (Fig. 4k). Serum cholesterol level was also significantly higher in *Gdf15*^−/−^ mice compared to control mice, while serum free fatty acid (FFA) and TG levels were slightly higher without statistical significance (Supplementary Fig. 4k). Cumulatively, our results indicate that GDF15 induction serves as a protective mechanism ameliorating AMLN diet-induced NASH and NASH-related metabolic deterioration.

### GDF15 suppresses expression of fibrogenic genes in HSCs and NASH livers

**Figure 5:**
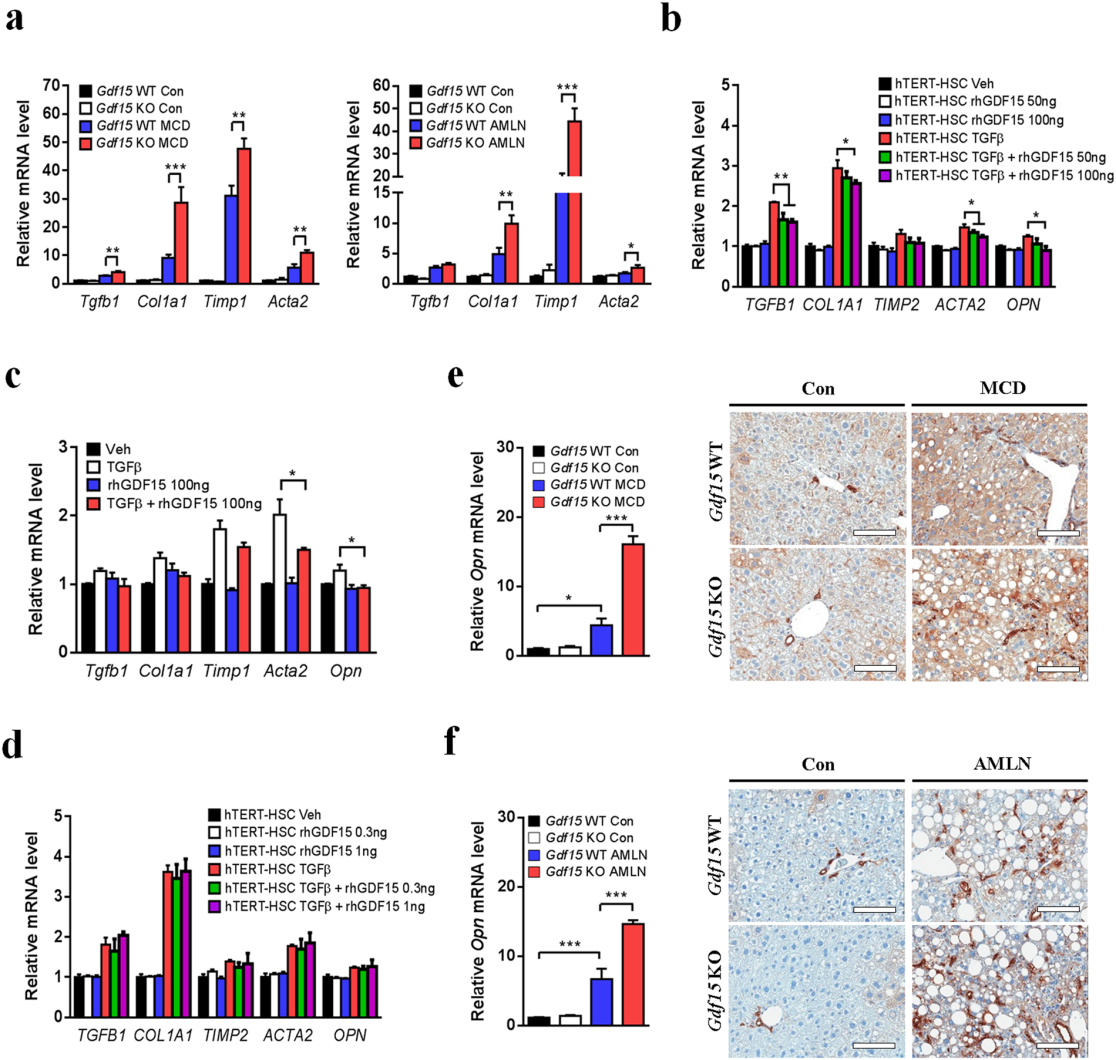
GDF15 suppresses expression of fibrosis-related genes and OPN in HSCs and NASH livers. (**a**) Relative fibrosis-related gene expression in the liver of *Gdf15*^–/–^ and control mice fed MCD diet for 8 weeks (n = 3–4) or AMLN diet for 30 weeks (n = 5–7). (**b**–**d**) Relative expression of fibrosis-related genes in hTERT-HSCs (**b,d**) and primary mouse HSCs (**c**) treated with TGFβ1 (2.5 ng/ml) in the presence or absence of GDF15 pretreatment. (**e,f**) Relative *Opn* mRNA levels and OPN immunostaining in the liver of *Gdf15*^–/–^ and control mice fed MCD for 8 weeks (**e**, n = 3–4) or AMLN diet for 30 weeks (**f**, n = 5–7). Scale bars, 100 μm. Data are means ± SEM. *p < 0.05, **p < 0.01, ***p < 0.001.

We next investigated the mechanism by which *Gdf15* deletion aggravates fibrosis in mice. We found that the expression of fibrosis-related genes (*Tgfb1*, *Col1a1*, *Timp1* and *Acta2*) was increased in the liver of *Gdf15*^−/−^ mice compared to control mice after MCD or AMLN diet feeding (Fig. 5a). To explain these *in vivo* findings, we investigated the *in vitro* effect of GDF15 on expression of fibrosis-related genes in HSCs, the major source of fibrogenic myofibroblasts, since HSCs play a key role in NASH-related fibrosis^32^. Treatment with 50 or 100 ng/ml recombinant GDF15 partially inhibited fibrosis-related gene expression in hTERT-HSCs and primary HSCs treated with TGFβ (Fig. 5b,c), although treatment with low doses (0.3–1 ng/ml) of GDF15 had no inhibitory effect on expression of fibrotic genes (Fig. 5d). These results suggest that treatment with pharmacological doses of GDF15 might directly exert anti-fibrogenic effect on HSCs. Several reports suggest that osteopontin (OPN) (also known as secreted phosphoprotein 1, SPP1) is induced in human subjects or mice with NASH^33,34^, and that OPN aggravates hepatic fibrosis in MCD diet-induced NASH mouse model^33^. Since OPN is upregulated in activated HSCs^35^, we next studied the effect of recombinant GDF15 on OPN expression in HSCs. Intriguingly, treatment with pharmacological doses of GDF15 suppressed OPN expression in TGFβ-treated hTERT-HSCs and primary HSCs (Fig. 5b,c).

When we investigated the change of OPN expression in the liver of *Gdf15*^−/−^ mice, expression of *Opn* gene was markedly increased in the liver of *Gdf15*^−/−^ mice after MCD or AMLN diet feeding (Fig. 5e,f). Furthermore, the number of OPN-positive cells was also higher in the liver of *Gdf15*^−/−^ mice compared to control mice after MCD or AMLN diet feeding (Fig. 5e,f). Although OPN is known to be a target gene of Hedgehog signaling pathway involved in MCD-induced NASH^34^, we did not observe increased expression of Hedgehog pathway-related genes in *Gdf15*^–/–^ mice after MCD or AMLN diet feeding (Supplementary Fig. 5a,b). Taken together, our findings suggest that GDF15 regulates expression of fibrosis-related genes and OPN in NASH.

### GDF15 overexpression alleviates diet-induced NASH and metabolic deterioration in mice

**Figure 6:**
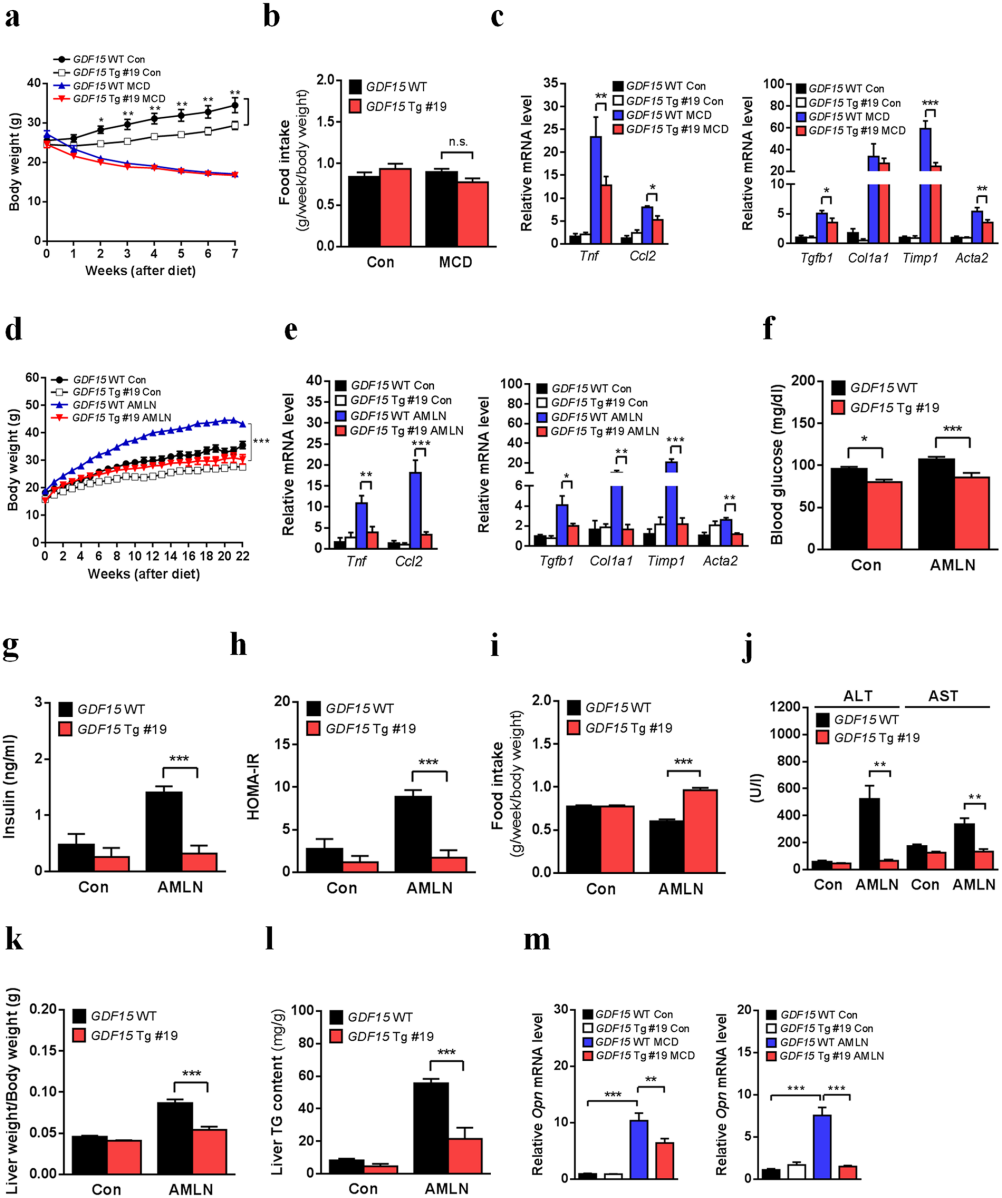
*GDF15* overexpression ameliorates NASH phenotypes and related metabolic deterioration in mice. (**a**–**c**) Body weight (**a**, n = 3–10), food intake adjusted for body weight (**b**, n = 3–6), relative inflammatory gene expression (left panel of **c**, n = 3–5) and relative fibrosis-related gene expression (right panel of **c**, n = 3–5) in *GDF15*-Tg line 19 and control mice fed MCD diet for 7 weeks. (**d**–**l**) Body weight (**d**, n = 4–11), relative inflammatory gene expression (left panel of **e**, n = 4–6), relative fibrosis-related gene expression (right panel of **e**, n = 4–6), fasting glucose level (**f**, n = 4–11), fasting insulin level (**g**, n = 4–11), HOMA-IR index (**h**, n = 4–11), food intake adjusted for body weight (**i**, n = 4–11), serum ALT/AST levels (**j**, n = 4–11), liver weight adjusted for body weight (**k**, n = 4–11) and liver TG content (**l**, n = 4–11) in *GDF15*-Tg line 19 and control mice fed AMLN diet for 22 weeks. (**m**) Relative hepatic *Opn* mRNA levels in *GDF15*-Tg line 19 and control mice fed MCD for 7 weeks (left, n = 3–5) or AMLN diet for 22 weeks (right, n = 4–6). Data are means ± SEM. *p < 0.05, **p < 0.01, ***p < 0.001.

Finally, to study whether GDF15 overexpression affects NASH development in the two dietary NASH models, we generated liver-specific *GDF15*-Tg mouse lines expressing human *GDF15* (Supplementary Fig. 6a). Among three *GDF15*-Tg mouse lines (14, 19 and 45) showing elevated human GDF15 protein level in serum (Supplementary Fig. 6b), we characterized NASH-related metabolic profile in two lines (14 and 45). We did not observe any differences in body weight and food intake between *GDF15*-Tg line 19 and control mice after MCD diet feeding, while *GDF15* overexpression reduced body weight of mice fed matched control diet (Fig. 6a,b). Importantly, *GDF15*-Tg line 19 mice had reduced expression of inflammatory and fibrotic genes compared to control mice fed MCD diet (Fig. 6c). When we employed AMLN diet model also, we found improved NASH and metabolic parameters in *GDF15*-Tg line 19 mice (Fig. 6d–h and Supplementary Fig. 7a–c) despite of increased food intake adjusted for body weight (Fig. 6i). Additionally, AMLN diet-fed *GDF15*-Tg line 19 mice exhibited decreases in liver enzyme levels (Fig. 6j), liver weight (adjusted for body weight) (Fig. 6k), and liver TG content (Fig. 6l), whereas these parameters were not significantly different between *GDF15*-Tg line 19 and control mice fed MCD diet (Supplementary Fig. 7d–f). Furthermore, *GDF15*-Tg line 19 mice had reduced hepatic expression of *Opn* gene in two dietary NASH models (Fig. 6m).

We next employed *GDF15*-Tg line 45 mice to confirm the effect of *GDF15* overexpression on NASH. When fed MCD diet, *GDF15*-Tg line 45 mice exhibited attenuated NASH phenotypes such as reduced expression of inflammatory and fibrosis-related genes (Supplementary Fig. 8a–f), similar to the results of *GDF15*-Tg line 19 mice fed MCD diet. Therefore, these data suggest that GDF15 overexpression leads to improvement of NASH in mice.

## Discussion

Growing evidence suggests that GDF15 is implicated in pathogenesis of various metabolic disorders such as obesity^21^, insulin resistance^11^, myocardial infarction^36^ and atherosclerosis^37,38^. Mice with overexpression of *GDF15* exhibited reduced adiposity and improved glucose clearance, which is probably attributable to enhancements of thermogenesis and lipid catabolism (lipolysis/β-oxidation), and attenuation of inflammation in adipose tissue, independent on food intake^21^. Furthermore, administration of recombinant GDF15 improves insulin resistance and glucose tolerance by decreasing food intake^39–42^ or by increasing thermogenesis, lipid catabolism or mitochondrial oxidative phosphorylation without change of food intake^11^. In addition, GDF15-induced beneficial effects on myocardial infraction, atherosclerosis and obesity-related insulin resistance are associated with its anti-inflammatory actions such as reduction of neutrophil recruitment to myocardial lesions^36^ and a decrease of macrophage infiltration in atherosclerotic lesions^37^ and adipose tissue^21^. Although NAFLD/NASH has been reported to be frequently associated with obesity or insulin resistance, to the best of our knowledge, the functional importance of GDF15 in NASH and related metabolic disorders has not been evaluated. Here, we demonstrated that hepatic GDF15 expression was increased in NASH mouse models. We also observed increased GDF15 expression in the liver of human NASH subjects, although large-scale studies are needed to evaluate the fundamental clinical importance of GDF15 in NASH. Importantly, we found that *Gdf15* deletion exacerbated hepatic steatosis, inflammation and fibrosis in NASH animal models using MCD or AMLN diet. Additionally, mice with deletion of *Gdf15* showed aggravated metabolic parameters such as increased body weight and elevated fasting glucose or insulin level after AMLN diet feeding. These results suggest that endogenous GDF15 plays a crucial role in attenuating NASH and NASH-related metabolic deterioration in mice. Additionally, we showed that *GDF15* overexpression led to alleviation of NASH phenotypes in mice. As a possible mechanism underlying protective effects of GDF15 on NASH-related fibrosis, we found that treatment with pharmacological doses of GDF15 directly suppressed expression of fibrosis-related genes and OPN in HSCs *in vitro*, although the expression of these genes in HSCs treated with physiological/pathological doses (0.3–1 ng/ml) of GDF15 was not changed. In addition, we found that *GDF15*-Tg mice showing supraphysiological serum GDF15 levels also had decreased expression of fibrotic genes and OPN in the liver. Notably, mice with deletion of *Gdf15* conversely showed increased expression of fibrosis-related gene in the liver. Therefore, all these results suggest that GDF15 could be a promising therapeutic target for treatment of NASH and NASH-related metabolic deterioration.

It has been reported that administration of GDF15 leads to decrease of body weight via reduction of food intake in mice^22^. GDNF family receptor α-like (GFRAL) has recently been identified as a novel receptor for GDF15 by four independent groups^39–42^. In these studies, GFRAL deletion abrogated the effect of GDF15 on body weight and food intake. Given that GFRAL is expressed solely in hindbrain neurons but not in peripheral tissues in mice^40,41^, these results suggest that exogenous GDF15 administration leads to resistance to diet-induced obesity via GFRAL-dependent anorexic action in brain. In contrast, we observed the beneficial effect of endogenous production or genetic overexpression of GDF15 on NASH progression independent of food consumption. These discrepancies in the effect of GDF15 on food consumption might be attributable to differences in experimental procedures (recombinant GDF15 administration *vs*. GDF15 overexpression), composition of diets (HFD *vs*. MCD or AMLN diet), duration of diet feeding (1–4 weeks *vs*. 7–30 weeks) or analytical methods of food intake measurement. Furthermore, previous some papers suggest that ALK5-TGFβRII complex also mediates the action of GDF15 in various tissues and cells^22,36^, suggesting the possibility that GDF15 might exert its metabolic effect via other receptors as well as GFRAL. Further studies will be necessary to elucidate the *in vivo* role of GFRAL or ALK5-TGFβRII complex in GDF15-induced improvement of NASH.

In the present study, we showed that *Gdf15* deletion led to aggravated hepatic steatosis, inflammation and fibrosis in mice after NASH diet, while mice overexpressing *GDF15* exhibited improvement of these phenotypes. We also observed increased fatty acid oxidation in hepatocytes and reduced macrophage recruitment as causes of GDF15-induced improvement of steatosis and inflammation. In addition, we showed direct anti-fibrotic effect of GDF15 on HSCs, which probably contributes to ameliorated NASH-related fibrosis in mice. The result is in line with a recent finding showing the anti-fibrotic effect of GDF15 on alcohol-induced liver injury^43^. However, other recent study has reported that GDF15 treatment increases fibrotic gene expression in HSCs^20^, which is inconsistent with our finding. In that paper, the effect of GDF15 on fibrosis was assessed in HSCs without TGFβ, a potent stimulator of fibrosis, while our papers studied the effect of GDF15 on HSCs in the presence of TGFβ. Furthermore, we observed that GDF15 alone treatment did not increase expression of fibrotic genes in HSCs. In parallel, *GDF15*-Tg mice did not exhibit increased fibrotic gene expression in the liver after matched control diet or regular chow diet. However, given that hepatic lipid accumulation and inflammation trigger fibrotic response in the progression of NASH, we cannot exclude the possibility that anti-fibrotic action of GDF15 is partly secondary to the reduced hepatic lipid accumulation and inflammation. Thus, further studies are needed to elucidate the mechanism underlying GDF15-mediated improvement of NASH-related fibrosis.

In conclusion, our results indicate that GDF15 is induced in NASH livers in a manner dependent on ER stress, which serves as a protective mechanism to attenuate hepatic steatosis, inflammation, fibrosis and metabolic deterioration. Therefore, our findings provide a new insight regarding the protective role of endogenous GDF15 in NASH and suggest an innovative therapeutic strategy for treatment of NASH or related metabolic disorders.

## Methods

### Animal experiments

*Gdf15*-knockout (*Gdf15*^−/−^) and *Gdf15*^+/+^ mice have been previously described^5^. Liver-specific *GDF15*-Tg mice were generated by microinjection of linearized pLiv7-*GDF15* into C57BL/6 zygotes (Macrogen Inc.). pLiv7-*GDF15* vector was generated by inserting human *GDF15* cDNA into pLiv-7 linearized with KpnI and XhoI. Three independent *GDF15*-Tg mouse founders (lines 14, 19 and 45) were established, and metabolic studies were performed employing two mouse lines (19 and 45). C57BL/6 mice were purchased from Orient-Bio Laboratory (Korea). Twelve-week-old *Gdf15*^−/−^ (or *GDF15*-Tg line 19), 16-week-old C57BL/6 or 20-week-old *GDF15*-Tg line 45 mice were fed either MCD diet (Envigo, Teklad Custom Diet, TD.90262) or matched control diet (TD.94149). As another dietary NASH mouse model, 5-week-old mice were fed AMLN diet comprising 40 kcal% fat, and 20 kcal% fructose and 2% cholesterol (Research Diets, D09100301) or matched control diet (10 kcal% fat) with no fructose or cholesterol (D09100304). Food intake was measured every week throughout the experimental periods. For *in vivo* ER stress experiment, mice were injected intraperitoneally with 1 mg/kg tunicamycin- or phosphate-buffered saline (PBS)-2% dimethyl sulfoxide (DMSO). For *in vivo* p53 inhibition, mice were administrated intraperitoneally with pifithrin-α (2.2 mg/kg, once per day for 7 days) or vehicle (5% DMSO, 5% Tween 80 and 90% PBS). For *in vivo* treatment with a chemical ER chaperone, mice were given 4-PBA (1 g/kg/day) in drinking water for 9 days. Male mice were used in all experiments. All animals were housed in individual ventilated cages (maximum of 5 mice per cage) under an environmentally controlled room (22 °C, 12 h light/dark cycle) with free access to food and water. All animals were maintained in a specific pathogen free (SPF) facility accredited by the Association for the Assessment and Accreditation of Laboratory Animal Care International (AAALAC). All animal experiments were approved by the Institutional Animal Care and Use Committee of Yonsei University Health System (IACUC of YUHS) and were conducted in accordance with the guidelines of IACUC of YUHS.

### Human NAFLD samples

Liver tissues were from 18 subjects (6 normal subjects, 6 subjects with simple steatosis and 6 subjects with NASH) who underwent hepatectomy at Severance Hospital due to primary liver cancer or metastatic (secondary) liver cancer. Clinical characteristics of all subjects are described in Supplementary Table 1. The non-tumor areas were isolated and used for RNA analysis. Liver histology was assessed by an experienced pathologist according to a diagnostic criteria^44^, and classified into three groups: normal, simple steatosis and NASH. Diagnostic criteria for NASH included the presence of biopsy-proven steatosis, ballooning, lobular inflammation and/or fibrosis without history of alcohol intake (<210 g/week for men and 140 g/week for women) and hepatitis B or C virus infection. Written informed consent was obtained from all subjects. This protocol and study were approved by the Institutional Review Board of Severance Hospital, Yonsei University College of Medicine. All experiments were performed in accordance with relevant guidelines and regulations.

### Transcriptome analyses

All transcriptomic data were from publicly available Gene Expression Omnibus (GEO) repository. To analyze the change of GDF15 expression in ASH, gene microarray data sets of the livers of human subjects with ASH (accession number GSE28619)^23^ were downloaded from the GEO database. Microarray data sets of the livers of hepatocyte-specific *Eif2ak3*-knockout mice^27^ and hepatocyte-specific *Ern1*-knockout mice^28^ from GEO repository were also used to evaluate the effect of PERK or IRE1α pathway on tunicamycin-induced GDF15 expression (GSE29929 and GSE40515). Heatmap analyses were conducted using GENE-E software (The Broad Institute).

### Reagents

4-PBA, pifithrin-α, tunicamycin, thapsigargin and DMSO were purchased from Sigma. Recombinant human TGFβ1 and human GDF15 were purchased from Peprotech and Biovision, respectively.

### Cell culture

HepG2 and Hepa1c1c7 cells were obtained from the American Type Culture Collection. CHOP MEFs were provided by R Kaufman. Human hepatic stellate cell line (hTERT-HSC) was kindly provided by D Brenner and WI Jeong^45^. Mouse Kupffer cell line (KUP5) was obtained from the RIKEN BioResource Center^46^. Primary mouse hepatocytes were isolated from 20-week-old *Gdf15* wild-type mice by collagenase digestion and Percoll density gradient method. Primary mouse HSCs were isolated from 20-week-old *Gdf15* wild-type mice using a previously described protocol with some modifications^47^. Briefly, the liver was perfused consecutively with EGTA, pronase (Sigma) and collagenase D (Sigma) solutions, and then was further digested with pronase/collagenase solution with DNase I (Sigma). HSCs were isolated by OptiPrep (Sigma) density gradient centrifugation. The purity of HSCs was assessed by retinol autofluorescence and appearance of lipid droplets. Cells were maintained in Dulbecco’s modified Eagle’s medium (DMEM) containing 10% fetal bovine serum and 1% antibiotics (penicillin-streptomycin-amphotericin B) at 37 °C in a humid atmosphere of 5% CO_2_. All cells were free of mycoplasma contamination. For analysis of GDF15 expression in an *in vitro* NASH model, Hepa1c1c7, KUP5 and hTERT-HSC cells were seeded at 6-well plates, incubated for 24 h and maintained in serum-free DMEM for 24 h. Cells were then incubated in MCD medium (WelGENE Inc.) or control DMEM for 18 h. To analyze fibrotic gene expression, serum-starved hTERT-HSCs and primary mouse HSCs were pre-treated with recombinant GDF15 (0.3, 1, 50 or 100 ng/ml) for 30 min, and then were incubated with recombinant TGFβ1 (2.5 ng/ml) for 18 h.

### RNA analysis

Total RNA was isolated from various cells or liver tissues using Hybrid-R or Ribospin^TM^ II Kit (GeneAll Biotechnology) according to the manufacturer’s instruction. cDNA was synthesized from 2 μg of total RNA using Moloney Murine Leukemia Virus (MMLV)-reverse transcriptase (Promega) and oligo(T) primer at 42 °C for 1 h. An aliquot (1/160 vol) of the cDNA was then subjected to PCR amplification using mouse or human gene-specific primers (Supplementary Table 2). Real-time RT-PCR was conducted using SYBR Green Master Mix (Takara) in a QuantStudio 3 Real-Time PCR machine (Applied Biosystems). The relative expression values of specific genes were normalized to that of *RPL32* (*Rpl32*) mRNA.

### Immunoblot analysis

Immunoblotting was performed as previously described^48^. Tissues and cultured cells were lysed in radio-immunoprecipitation assay (RIPA; 50 mM Tris-HCl, pH 7.4, 150 mM NaCl, 0.25% sodium deoxycholate, 1 mM EDTA, 1% (v/v) NP-40) buffer supplemented with 1 mM PMSF and a protease inhibitor cocktail (Roche) for 30 min on ice. Protein extracts were obtained by centrifugation at 18,000 × g for 20 min at 4 °C. Protein concentrations were measured by the Bradford method using Bio-Rad Protein Assay Dye Reagent (Bio-Rad). Proteins in the lysates were separated on SDS/PAGE gels (7–12%) or NuPAGE Bis-Tris Gels (ThermoFisher), and transferred to PVDF membranes (Millipore). Membranes were then incubated with antibodies against HSP90 (sc-7947; Santa Cruz Biotechnology, 1:5000 dilution), ATF4 (#11815; Cell Signaling, 1:1000 dilution), total eIF2α (#9722; Cell Signaling, 1:2000 dilution), phospho-eIF2α (#3597; Cell Signaling, 1:1000 dilution), CHOP (sc-7351; Santa Cruz Biotechnology, 1:1000 dilution), GDF15 (sc-10607; Santa Cruz Biotechnology, 1:1000 dilution) or p53 (OP03; Millipore, 1:1000 dilution) in Tris-buffered saline with 0.05% Tween-20 (TBST) containing 5% (w/v) non-fat dry milk. After incubation of membranes with horseradish peroxidase-conjugated secondary antibodies, immunoreactive protein bands were visualized using EZ-Western Lumi Femto ECL solution (DoGen).

### Histology and staining analysis

Liver tissues were harvested and immediately fixed with 10% neutral buffered formalin (Sigma) to make paraffin-embedded blocks. Hematoxylin and eosin (H&E) staining, Sirius red staining, Masson’s trichrome staining and immunohistochemistry were conducted using paraffin-embedded tissue sections. For immunohistochemistry, liver sections were stained with antibodies against αSMA (ab5694; Abcam, 1:150 dilution), F4/80 (ab6640; Abcam, 1:100 dilution), osteopontin (AF808; R&D Systems, 1:100 dilution) or CLEC4F (AF2784; R&D Systems, 1:200 dilution). For quantification for immunohistochemical staining, stained slides were scanned using an Aperio Scanscope AT Turbo instrument (Aperio Technologies) and analyzed using ImageScope software (Aperio Technologies). For all analyses, 20 randomly chosen fields from at least 4–5 sections per group were quantified using NIH ImageJ software.

### Blood chemistry and metabolite analysis

Glucose levels in blood samples obtained from tail vein were measured with an Accu-Check glucometer (Roche). Serum insulin level was determined with a Mouse Insulin ELISA Kit (Shibayagi). Serum ALT, AST, TG and cholesterol levels were measured with a Fuji Dri-Chem 3500 biochemistry analyzer (Fujifilm). Serum GDF15 level was determined using a Mouse GDF-15 DuoSet ELISA Kit (R&D Systems) or a Human GDF-15 Quantikine ELISA Kit. Serum FFA level was determined using a SICDIA NEFAZYME Kit (Shinyang Chemical). Liver TG content was measured using a Triglyceride Kit (GPO-Trinder, Sigma). HOMA-IR index was calculated according to the following formula: [fasting insulin (μlU/ml) × fasting glucose (mg/dl)]/405.

### Statistical analysis

All values are expressed as means ± SEM. Statistical significance was tested with an unpaired two-tailed Student’s t test to compare two groups and one-way analysis of variance (ANOVA) with a post-hoc Newman-Keuls test to compare multiple groups. All analyses were performed using GraphPad Prism Version 6 software (La Jolla, CA, USA). *P* values less than 0.05 were considered to indicate statistically significant differences.

### Data availability

All data generated or analyzed during this study are available from the corresponding author on reasonable request.

## Electronic supplementary material

Supplementary information
