# Supplementary material for: Growth differentiation factor 15 ameliorates nonalcoholic steatohepatitis and related metabolic disorders in mice
Source: Sci Rep. 2018 May 1;8:6789. doi: 10.1038/s41598-018-25098-0 (PMC5931608; doi:10.1038/s41598-018-25098-0)
Supplement: Supplementary file 1 — Supplementary information [file 41598_2018_25098_MOESM1_ESM.pdf]

Supplementary Information

Growth differentiation factor 15 ameliorates nonalcoholic steatohepatitis and related metabolic disorders in mice

Kook Hwan Kim, Seong Hun Kim, Dai Hoon Han, Young Suk Jo, Yong-ho Lee, Myung-Shik Lee

<CONTENTS>

Supplementary Tables

Supplementary Figures

Supplementary Table 1

Clinical characteristics of human subjects used in this study.

Group	No	Gender	Age	BMI	Diagnosis	Liver metastasis
Control	1	F	57	16.78	Rectal cancer	Y
	2	M	75	20.76	AGC	Y
	3	M	34	24.36	RSC, FAP	Y
	4	M	57	26.89	Rectal cancer	Y
	5	F	47	17.12	Sigmoid cancer	Y
	6	F	48	22.43	RSC	Y
Simple steatosis	1	M	38	18.57	Rectal cancer	Y
	2	F	49	21.88	Colon cancer	Y
	3	M	58	23.53	Rectal cancer	Y
	4	M	66	23.57	Colon cancer	Y
	5	F	52	27.34	Colon cancer	Y
	6	F	62	25.68	Colon cancer	Y
NASH	1	M	65	24.84	Rectal cancer	Y
	2	M	59	26.57	HCC	N
	3	M	76	24.79	HCC	N
	4	M	67	27.31	HCC	N
	5	M	78	20.83	HCC	N
	6	M	55	25.05	Sigmoid cancer	Y

*AGC (advanced gastric cancer)
RSC (Rectosigmoid junction cancer)
FAP (Familial adenomatous polyposis)
HCC (Hepatocellular carcinoma)

Supplementary Table 2

Primers used in real-time RT-PCR (5’ to 3’).

Gene	Forward	Reverse
mAcaca	CCAGGCCATGTTGAGACGCT	ATCACAGAGCGGACGCCATC
mAcadl	GGGAATGAAAGCTCAGGACA	AGAATCCGCATTAGCTGCAT
mAcadm	AGGTTTCAAGATCGCAATGG	CTCCTTGGTGCTCCACTAGC
hACTA2	GGCAAGTGATCACCATCGGA	GTGGTTTCATGGATGCCAGC
mActa2	ATGCTAACAACGTCCTGTCA	AGAGTACTTGCGTTCTGGAG
mCcl2	CCCAATGAGTAGGCTGGAGA	TCTGGACCCATTCCTTCTTG
mCd36	TCTTTGAGCCTTCACTGTCT	CTCAATGTCCGAGACTTTTC
mChop	GAAGCCTGGTATGAGGATCT	ACTGACCACTCTGTTTCCGT
mClec4F	GACCATTGGATTGGGCTCAC	CTCGCTCTCCGTTCCTATGT
hCOL1A1	GCTTCACCTACAGCGTCACT	AAGCCGAATTCCTGGTCTGG
mCol1a1	GCTCCTCTTAGGGGCCACT	CCACGTCTCACCATTGGGG
mCpt1a	CCGATCATGGTTAACAGCAA	TGCAGCAGAGATTTGGCATA
mFasn	CTCCGTGGACCTTATCACTA	CTGGGAGAGGTTGTAGTCAG
hGDF15	CTCCAGATTCCGAGAGTTGC	CACTTCTGGCGTGAGTATCC
mGdf15	AGTGTCCCCACCTGTATCG	TGTCCTGTGCATAAGAACCA
mGli1	CGGCCAATCACAAATCAGGC	CACAGCTGGGGTTGGTATCC
mGli2	CTGAGCCCAAACATTGTCAG	CTGATGGGATGACAGAAGGT
mIhh	GGCCATCACTCAGAGGAGTC	TTTCGGTCACGGTCTGAGGT
mIl6	CAACCACGGCCTTCCCTACT	TTCTGCAAGTGCATCATCGTTGT
mLipc	AGCCTACTTTTACCATGTCG	AGAGTGGTGAGGTTCTATGC
mLipe	AAGGACTTGAGCAACTCAGA	TTGACTATGGGTGACGTGTA
mLpl	ATGGAGAGCAAAGCCCTGCTC	ATACTCAAAGTTAGGCCCAGC
mMgll	GACGGACAGTACCTCTTTTG	AGAAAAGTAGGTTGGCCTCT
hOPN	AGAGTGCTGAAACCCACAGC	GGAATTCACGGCTGACTTTGG
mOpn	AGGACAACAACGGAAAGGGC	ATCCGACTGATCGGCACTCT
mPnpla2	CATGATGGTGCCCTATACTC	GTGAGAGGTTGTTTCGTACC
mPpara	GGATGTCACACAATGCAATTCG	TCACAGAACGGCTTCCTCAGGT
mPparg	ATCCCTGGTTTCATTAACCT	GCTCCATAAAGTCACCAAAG
mPtch1	ATGAGAGCTACCCTGAGACT	TCACATTCCACGTCCTGTAG
hRPL32	CATCCGGCACCAGTCAGACC	TGTGAGCGATCTCGGCACAG

mRpl32	CAGTCAGACCGATATGTGAA	TAGAGGACACATTGTGAGCA
mScd1	TGGAAATGCCTTTGAGATGG	CCAGCCAGCCTCTTGACTAT
mSrebf1	TGCGGCTGTTGTCTACCATA	TGCTGGAGCTGACAGAGAAA
hTGFB1	TGGTGGAAACCCACAACGAA	GAGCAACACGGGTTCAGGTA
mTgfb1	TGAGTGGCTGTCTTTTGACG	GGTTCATGTCATGGATGGTG
mTimp1	CTTGGTTCCCTGGCGTACTC	ACCTGATCCGTCCACAAACAG
hTIMP2	GCAACAGGCGTTTTGCAATG	GTGATGTGCATCTTGCCGTC
mTnf	GCTACGACGTGGGCTACAG	CCCTCACACTCAGATCATCTTCT

Supplementary Figures

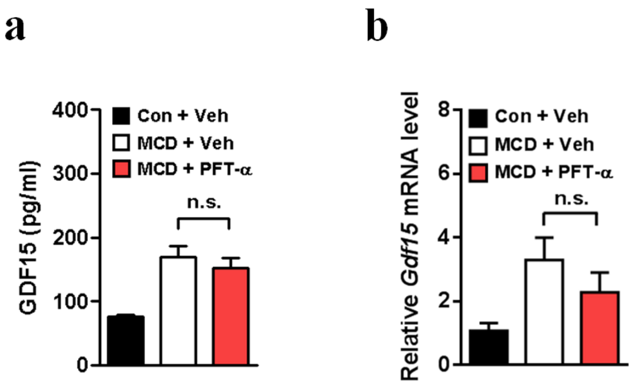

Supplementary Figure 1. The effect of p53 inhibitor on GDF15 expression in C57BL/6 mice fed MCD diet. (**a**) Serum GDF15 level in MCD diet-fed C57BL/6 mice after treatment with pifithrin-α (PFTα, 2.2 mg/kg/day) or Veh for 7 days (n = 4). (**b**) Relative hepatic *Gdf15* mRNA expression in MCD diet-fed C57BL/6 mice after treatment with pifithrin-α (PFTα, 2.2 mg/kg/day) (n = 4). Data are means ± SEM. n.s. indicates not significant.

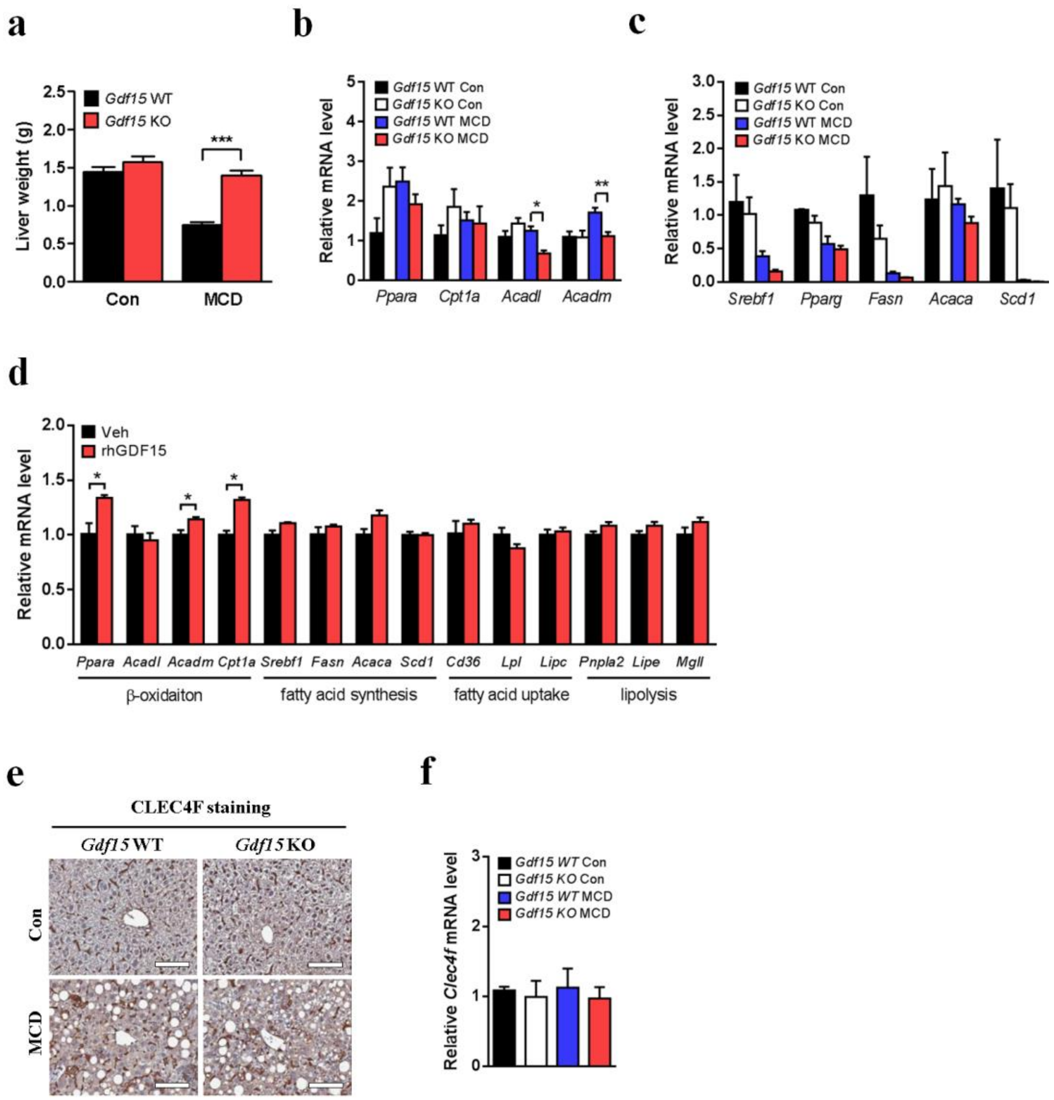

Supplementary Figure 2. Effects of *Gdf15* deletion and exogenous GDF15 on NASH and expression of lipid metabolism-related genes. (**a**–**c**) Liver weight (**a**, n = 5-7), and relative expression of β-oxidation-related genes (**b**, n = 3-4) or fatty acid synthesis-related genes (**c**, n = 3-4) in the liver of *Gdf15*$^{-/-}$ and control mice fed MCD or matched control diet. (**d**) Relative expression of hepatic lipid metabolism-related genes in primary mouse hepatocytes treated recombinant human GDF15 (rhGDF15) (n = 3). (**e**, **f**) CLEC4F immunohistochemistry of the liver sections (**e**) and relative *Clec4f* mRNA expression in the liver tissues (**f**, n = 3-4) from *Gdf15*$^{-/-}$ and control mice fed MCD or matched control diet. Scale bars, 100 μm. Data are means ± SEM. * $p < 0.05$, ** $p < 0.01$, *** $p < 0.001$.

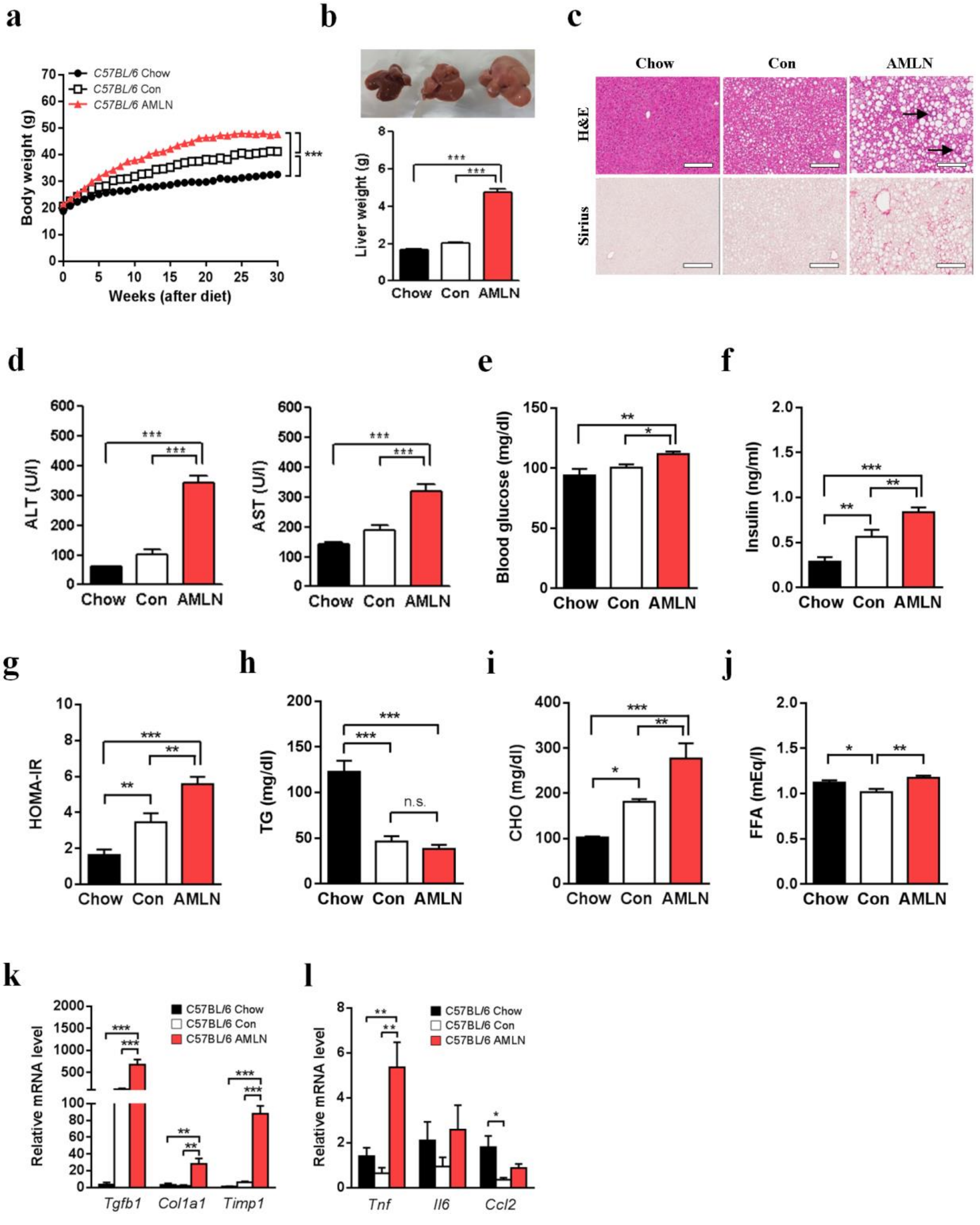

Supplementary Figure 3. NASH and metabolic parameters in C57BL/6 mice fed AMLN diet

for 30 weeks. (**a**) Body weight (n = 7). (**b**) Gross image and weight of the liver (n = 7). (**c**) H&E and Sirius red staining of liver sections. Arrows indicate inflammatory loci. (**d**) Serum ALT/AST levels (n = 7). (**e**) Fasting blood glucose level (n = 7). (**f**) Fasting serum insulin level (n = 7). (**g**) HOMA-IR index (n = 7). (**h**) Serum TG level (n = 7). (**i**) Serum cholesterol (CHO) level (n = 7). (**j**) Serum free fatty acid (FFA) level (n = 7). (**k**) Relative expression of fibrosis-related genes in the liver tissue (n = 5-7). (**l**) Relative expression of inflammatory genes in the liver tissue (**n** = 5-7). Scale bars, 200 μm. Data are means ± SEM. * $p < 0.05$, ** $p < 0.01$, *** $p < 0.001$. n.s. indicates not significant.

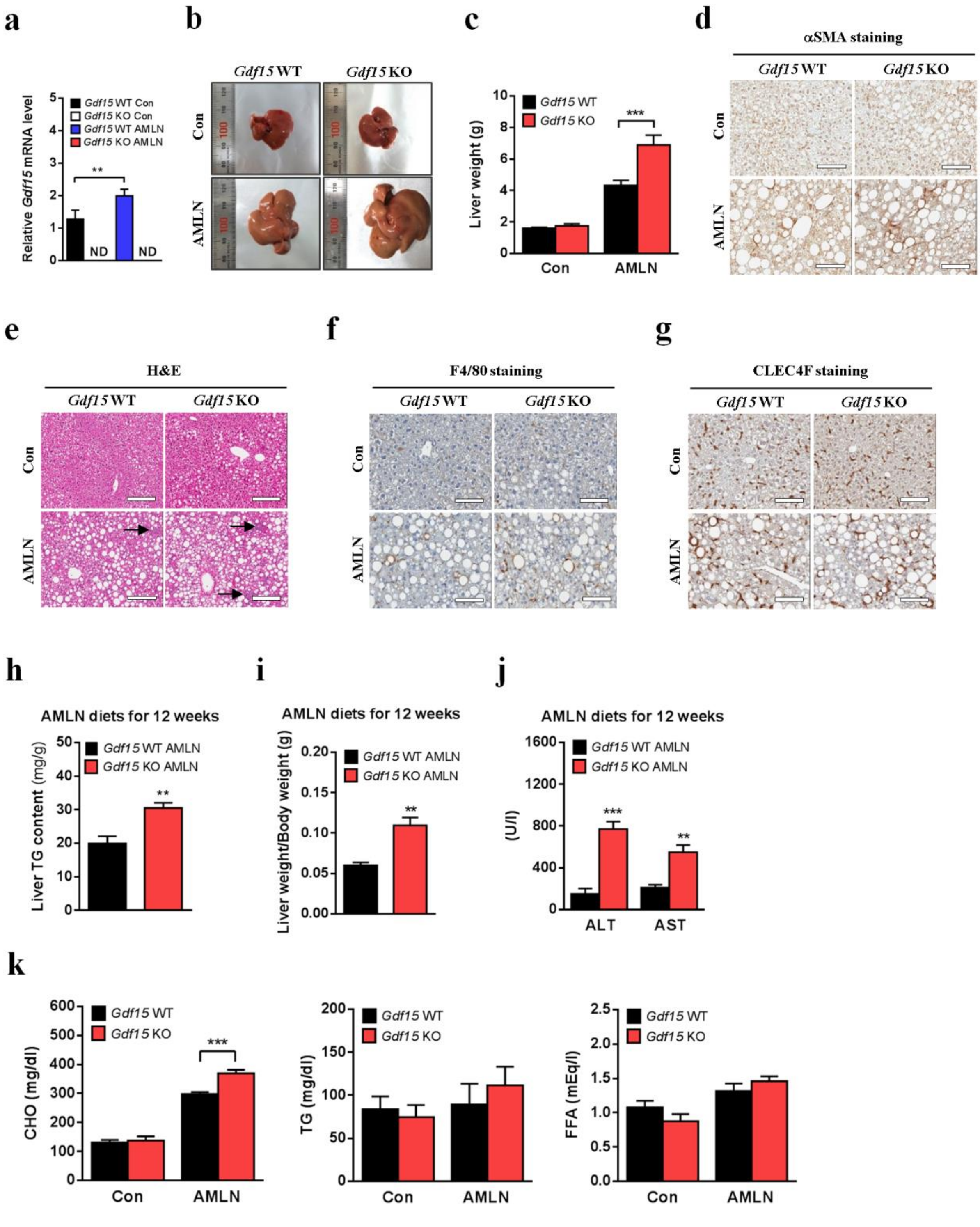

Supplementary Figure 4. Effects of *Gdf15* deletion on NASH after AMLN diet feeding. (**a**–**c**) Relative hepatic *Gdf15* mRNA expression (**a**, n = 5-7), gross liver image (**b**) and liver weight (**c**,

n = 7-12) of *Gdf15*$^{-/-}$ and control mice fed AMLN diet for 30 weeks. ND indicates not detected. (**d**–**g)** αSMA immunostaining (**d**), H&E staining (**e**), F4/80 immunostaining (**f**) and CLEC4F immunohistochemistry (**g**) of the liver sections from *Gdf15*$^{-/-}$ and control mice fed AMLN diet for 30 weeks. Arrows indicate inflammatory loci. (**h**–**j**) Liver TG content (**h**), liver weight adjusted for body weight (**i**) and serum ALT/AST levels **(j)** of *Gdf15*$^{-/-}$ and control mice fed AMLN diet for 12 weeks (n = 4-5). (**k**) Serum CHO level (left panel of **k**, n = 6-9), serum TG level (middle panel of **k**, n = 6-9) and serum FFA level (right panel of **k**, n = 6-9) of *Gdf15*$^{-/-}$ and control mice fed AMLN diet for 30 weeks. Scale bars for H&E staining, 200 μm. Scale bars for αSMA, F4/80 and CLEC4F immunostaining, 100 μm. Data are means ± SEM. ** $p < 0.01$, *** $p < 0.001$.

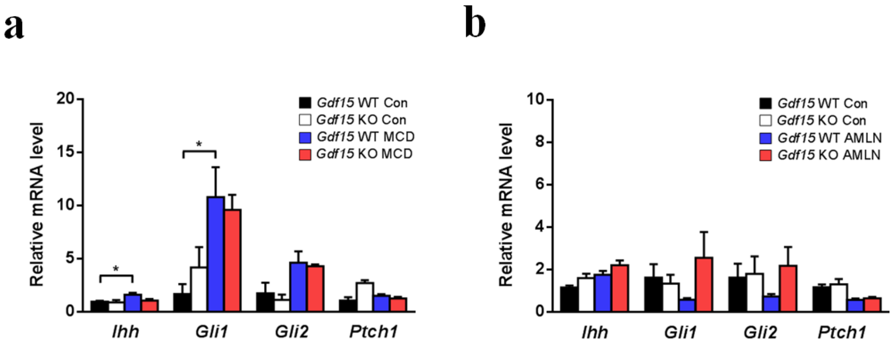

Supplementary Figure 5. Gene expression of Hedgehog pathway in the livers of *Gdf15*$^{-/-}$ mice fed MCD or AMLN diet. (**a**) Relative expression of Hedgehog signaling-related genes in the livers of *Gdf15*$^{-/-}$ and control mice fed MCD (n = 3-4). (**b**) Relative expression of Hedgehog signaling-related genes in the livers of *Gdf15*$^{-/-}$ and control mice fed AMLN diet (n = 5-7). Data are means ± SEM. * $p < 0.05$.

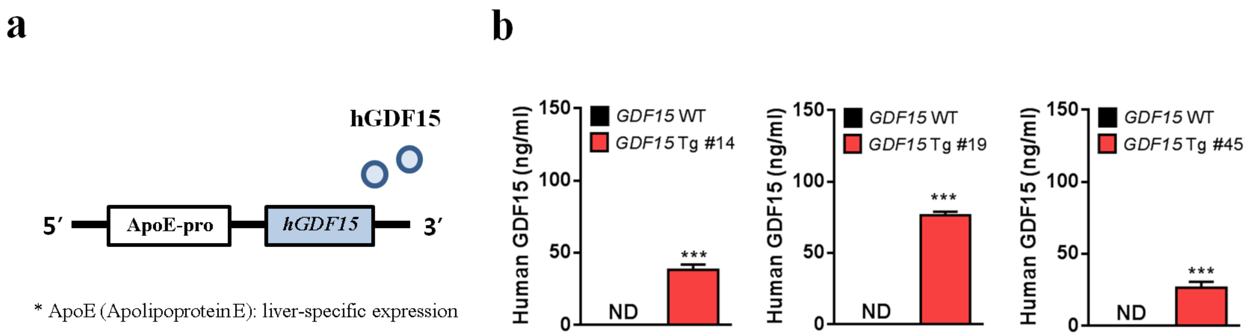

Supplementary Figure 6. Generation of *GDF15*-Tg mice. (**a**) Schematic diagram of construct driving overexpression of human *GDF15* under the control of the apolipoprotein promoter (ApoE-pro) that was used to generate *GDF15*-Tg mice. (**b**) Serum human GDF15 levels in 20-week-old *GDF15*-Tg line 14 (left panel, n = 2-5), 8-week-old *GDF15*-Tg line 19 (middle panel, n = 5-11), and 20-week-old *GDF15*-Tg line 45 mice (right panel, n = 6-7). ND indicates not detected.

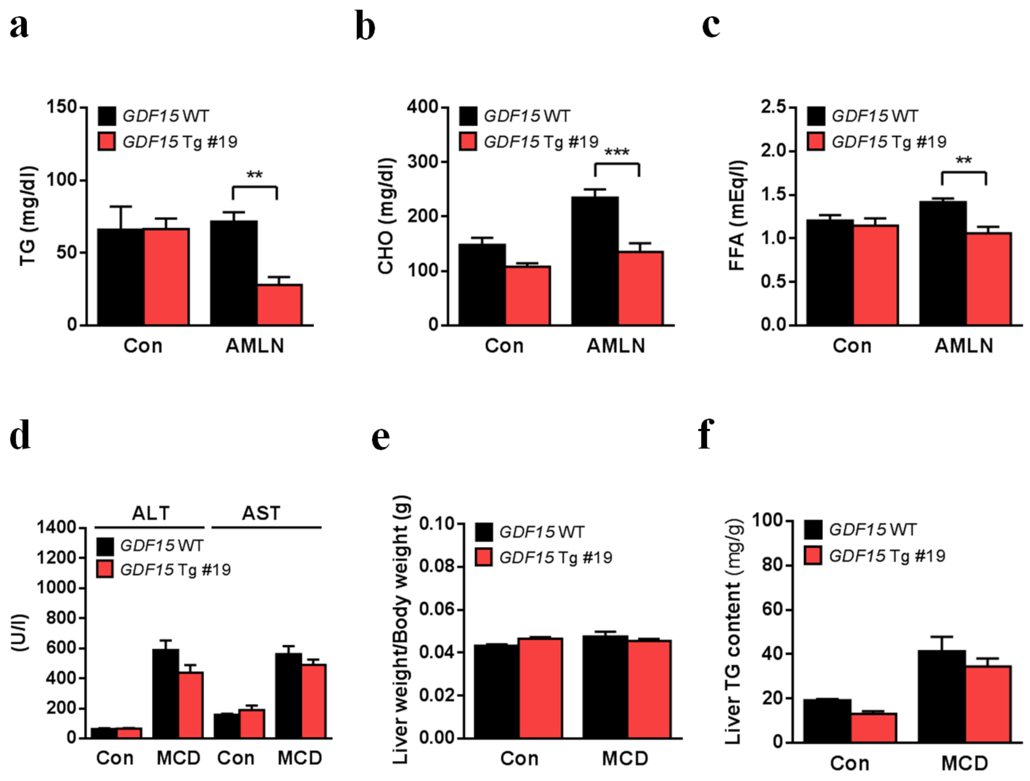

Supplementary Figure 7. NASH-related metabolic profile in *GDF15*-Tg mouse line 19. (**a**–**c**) Serum TG level (**a**), serum CHO level (**b**) and serum FFA level (**c**) in *GDF15*-Tg line 19 mice and control mice fed AMLN diet for 22 weeks (n = 4-11). (**d**–**f**) Serum ALT/AST levels (**d**, n = 3-10), liver weight adjusted for body weight (**e**, n = 3-6) and liver TG content (**f**, n = 3-6) in *GDF15*-Tg line 19 mice and control mice fed MCD diet for 7 weeks. Data are means ± SEM. ** $p < 0.01$, *** $p < 0.001$.

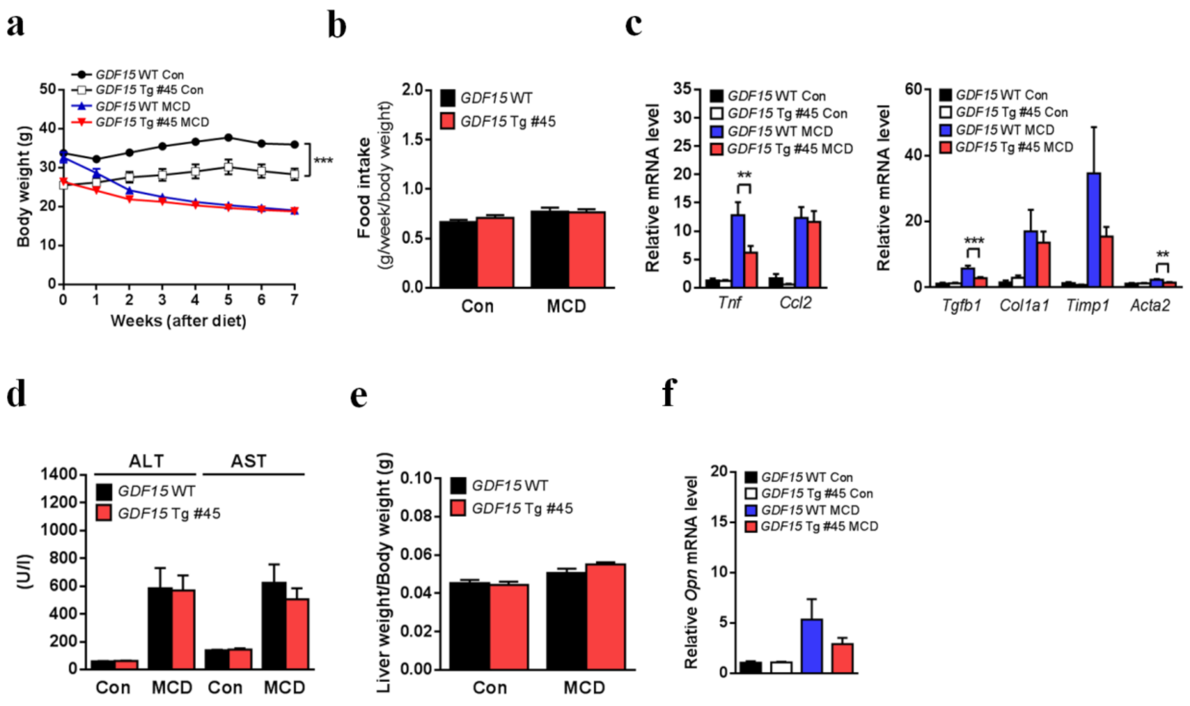

Supplementary Figure 8. NASH-related metabolic profile in *GDF15*-Tg mouse line 45 fed MCD diet for 7 weeks. (**a**) Body weight. (**b**) Food intake adjusted for body weight. (**c**) Relative inflammatory gene expression (left panel) and relative fibrosis-related gene expression (right panel). (**d**) Serum ALT/AST levels. (**e**) Liver weight adjusted for body weight. (**f**) Relative *Opn* mRNA expression in the liver tissue. Data are means ± SEM. ** $p < 0.01$, *** $p < 0.001$. ($n = 6$-7).

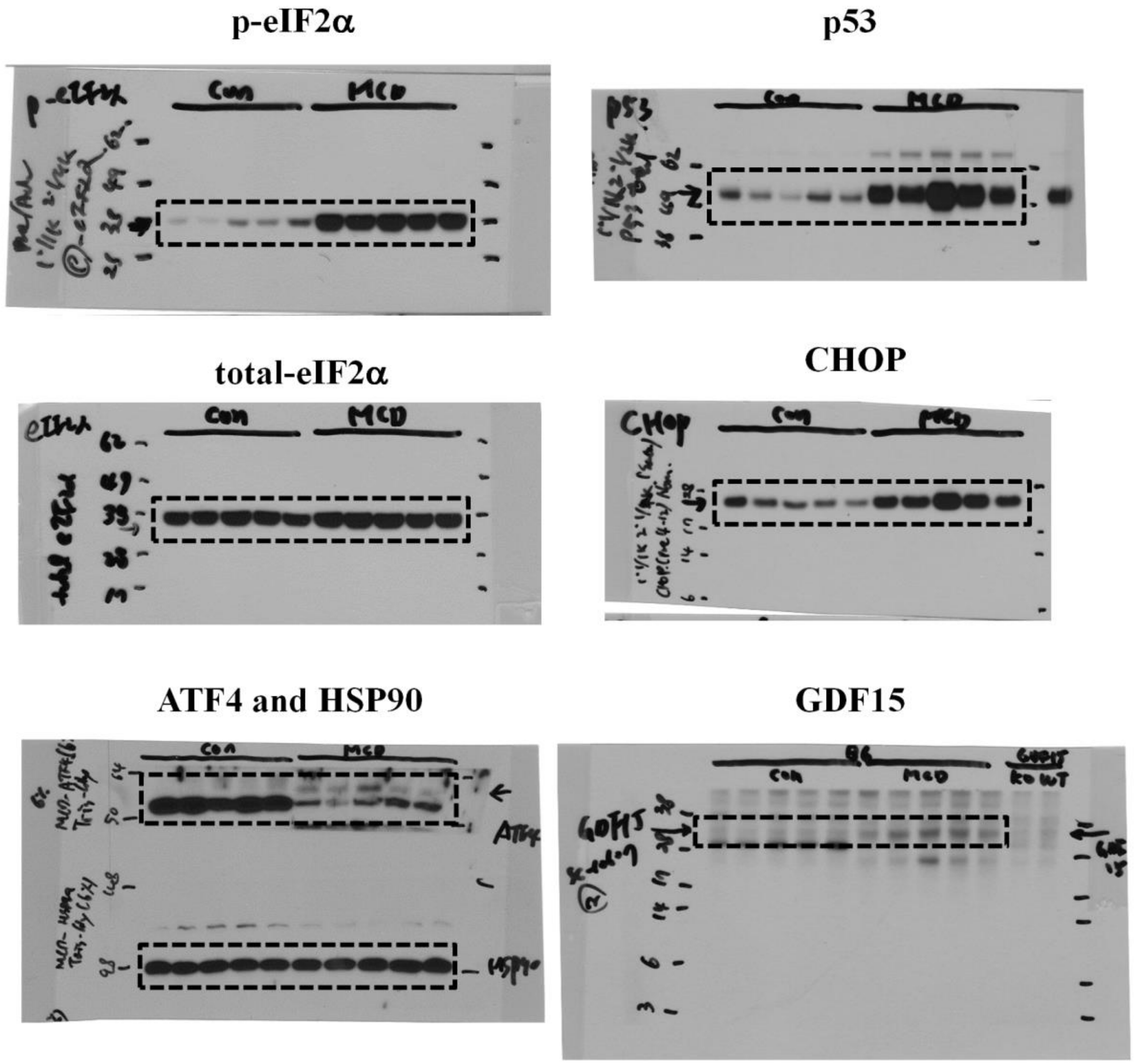

Supplementary Figure 9. Full scan original films of Western blot analysis in Figure 2a.
